# Use of Bacteriocins and Bacteriocinogenic Beneficial Organisms in Food Products: Benefits, Challenges, Concerns

**DOI:** 10.3390/foods11193145

**Published:** 2022-10-10

**Authors:** Svetoslav Dimitrov Todorov, Igor Popov, Richard Weeks, Michael Leonidas Chikindas

**Affiliations:** 1 ProBacLab, Laboratório de Microbiologia de Alimentos, Departamento de Alimentos e Nutrição Experimental, Faculdade de Ciências Farmacêuticas, Universidade de São Paulo, São Paulo 05508-000, SP, Brazil; 2 Center for Agrobiotechnology, Don State Technical University, 344002 Rostov-on-Don, Russia; 3 Health Promoting Naturals Laboratory, School of Environmental and Biological Sciences, Rutgers State University, New Brunswick, NJ 08904, USA; 4 Department of General Hygiene, I.M. Sechenov First Moscow State Medical University, 119991 Moscow, Russia

**Keywords:** antimicrobial peptides, bacteriocins, lactic acid bacteria, food safety, sustainable

## Abstract

This review’s objective was to critically revisit various research approaches for studies on the application of beneficial organisms and bacteriocins as effective biopreservatives in the food industry. There are a substantial number of research papers reporting newly isolated bacterial strains from fermented food products and their application as potential probiotics, including partial characterization of bacteriocins produced by these microorganisms. Most of these studies follow scientific community-accepted standard procedures and propose various applications of the studied strains and bacteriocins as potential biopreservatives for the food industry. A few investigations go somewhat further, performing model studies, exploring the application of expressed bacteriocins in a designed food product, or trying to evaluate the effectiveness of the studied potential probiotics and bacteriocins against foodborne pathogens. Some authors propose applications of bacteriocin producers as starter cultures and are exploring in situ bacteriocin production to aid in the effective control of foodborne pathogens. However, few studies have evaluated the possible adverse effects of bacteriocins, such as toxicity. This comes from well-documented reports on bacteriocins being mostly non-immunogenic and having low cytotoxicity because most of these proteinaceous molecules are small peptides. However, some studies have reported on bacteriocins with noticeable cytotoxicity, which may become even more pronounced in genetically engineered or modified bacteriocins. Moreover, their cytotoxicity can be very specific and is dependent on the concentration of the bacteriocin and the nature of the targeted cell. This will be discussed in detail in the present review.

## 1. Introduction

**Figure 1:**
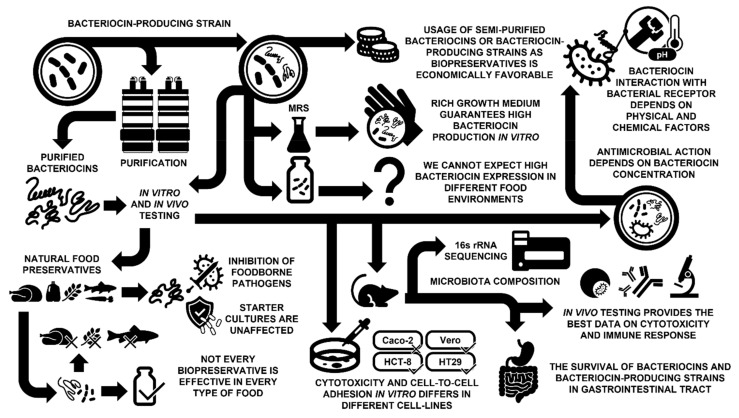
Keystone points in evaluation of potential application of bacteriocins as powerful antimicrobials in biopreservation of food commodities and/or new generation of drugs.

The search for optimal manufacturing conditions (raw materials and their processing, storage, distribution of the final product, etc.) has always been an objective of the food industry. Producing food commodities at an optimal price, with extended shelf life, covering consumer demand for healthier ingredients, and without chemical preservatives can be supported by using antimicrobials of microbial origin, such as bacteriocins. Bacteriocins are easy to produce, generally safe, and specific in their mode of action, qualities that should make them perfect antimicrobials for the food industry. However, are bacteriocins really an answer to the demand for safe, nontoxic, and effective antimicrobial preservatives for the food industry? Since the discovery of bacteriocins, the search for bacteriocins with potential applications in the food industry has been the subject of more than 13276 papers (www.scopus.com, accessed 29 August 2022). European regulatory authorities authorized the use of nisin in 1983 [1] for application in some food products as an effective biopreservative for the control of spoilage organisms. Five years later, the FDA approved the use of nisin in the USA (FDA 21CFR184.1538; https://www.accessdata.fda.gov/scripts/cdrh/cfdocs/cfcfr/cfrsearch.cfm?fr=184.1538, accessed on 25 August 2022). Worldwide, nisin’s approved applications cover a wide variety of products with often very different permitted levels of use (for more information, see Todorov, Franco, Tagg [2]). However, out of the many bacteriocins that have been investigated, only nisin and later pediocin PA-1 (for reviews, see Silva et al. [3] and Soltani et al. [4]) are approved for use in the food industry as partially purified bacteriocins. What is the reason for such limited use, with only two bacteriocins selected from a large cohort of potent antimicrobial bacteriocins? Is it because many (if not most) isolated and characterized bacteriocins are not suited for large-scale production and application? Or, perhaps, because other bacteriocins are less potent than those already utilized by the food industry? Are there strong lobbying and industry pressures behind the production of nisin? Or is there more scrutiny in approving new food additives so that novel bacteriocins can no longer meet regulatory requirements? Whatever the answer is, research on the isolation, characterization, and proposal of new bacteriocins as potentially effective biopreservatives is a growing scientific trend in need of common guidelines and a consensus on appropriate methods and reporting standards (Figure 1).

Moreover, in addition to nisin and pediocin PA-1, some other bacteriocins were applied as part of multicomponent commercial preparations, recently reviewed by Chikindas et al. [5], most of them associated with the application of nisin A (BioSate^TM^, Chr. Hansen) produced by *Lactococcus lactis* subsp. *lactic* BS-10, applied for the control of different cheeses and the prevention of off-flavors and late blowing associated with clostridia contaminations; undefined bacteriocins (HOLDBACK^TM^, Dupont), produced by *Propionibacterium freudenreichii* subsp. *shermanii* DSM 706 and *Lacteicaseibacillus rhamnosus* DSM 7061, for the inhibition of mold and psychrotrophs in cottage cheeses; Sakacin A and pediocin PA-1/AcH (Bactoferm^TM^ F-LC, Chr. Hansen), produced by *Latilactobacillus curvatus*, *Pediococcus acidilactici*, and *Staphylococcus xylosus* strains, for the control of *Listeria monocytogenes* and as a meat fermentation starter; plantaricin and carnocin (ALCMix1, Danisco DuPont), produced by *Lactiplantibacillus plantarum* and *Staphylococcus carnosus*, for the control of listerial contaminants in fermented sausages and cooked ham; leucocin (Bactoferm^TM^ B-SF-43, Chr. Hansen), produced by *Leuconostoc carnosum*, for the control of listeria in vacuum and modified atmosphere stored meat products; and sakacin (Bactoferm^TM^ B-2 and Bactoferm^TM^ B-FM, Chr. Hansen), produced by *Latilactobacillus sakei* (alone or in combination with *Staphylococcus xylosus*), for the control of listeria in stored or fresh meat products [5].

Therefore, we aimed to be critical in discussing different research approaches for performing studies on the application of bacteriocinogenic lactic acid bacteria (LAB) and bacteriocins as effective biopreservatives for the food industry.

## 2. Bacteriocins: What Good Do They Do for Us in Food Products?

Production of antimicrobial metabolites, including bacteriocins, is an evolutionary response resulting in the creation of effective survival mechanisms so microbial species can compete, communicate, and protect themselves in multimicrobial environments [6]. Bacteriocins are “bacterially produced peptides that are active against other bacteria and against which the producer has a specific immunity mechanism” [7]. The functional role of bacteriocins in natural environments (soil, water reservoirs, etc.) is not yet fully understood. We often refer to their antimicrobial properties as their key feature, playing a role in protecting ecological niches and competing with other “neighboring” microorganisms (for a recent in-depth review, see Heilbronner et al. [8]). However, the signaling role of bacteriocins and their involvement in *quorum sensing* processes may be their primary role in the life of a microbial community [5].

Bacteriocins from LAB are peptides (small proteins) with specific chemical and physical characteristics that influence their specific interactions with targeted cells [9]. Generally, bacteriocins are active against organisms closely related to the producing species [2]. However, a few examples from the last two decades provide evidence that some bacteriocins produced by LAB can be inhibitory against specific Gram-negative bacterial species, yeasts and fungi [2], *Mycobacterium* [10,11], and even some viruses [12,13]; for a review with emphasis on a proposed strategy for the use of bacteriocinogenic probiotics, see Tiwari et al. [14]. Such reports are an interesting contribution to our knowledge of bacteriocins; however, it must be noted that, in several cases, these nonspecific activities may be a consequence of the combined action between bacteriocins and some elements of the culturing environment [15]. Even if this is the case, the combined application of bacteriocins and additional synergetic factors against clinically relevant pathogens and spoilage microorganisms merits scientific attention and the further development of effective therapeutic and biopreservative agents (see reviews Cavera et al. [16], Chikindas et al. [5], and Arthur et al. [17]).

The successful application of bacteriocins in the food industry as a factor for food safety can be associated with the extension of shelf life and protection against foodborne pathogens, especially in nonrefrigerated products. Consequently, there is a significantly reduced risk of transmission of pathogens and spoilage microorganisms from these products. In addition to reducing the number of foodborne pathogens in food products, bacteriocins significantly reduce economic losses associated with food spoilage, outbreaks, and recalls. It is important to mention that bacteriocins are defined as natural antimicrobial agents and, thus, fit into the consumers’ growing requests for fewer chemical preservatives and treatments and a growing trend toward more natural food products [4].

Nisin is one of the most extensively studied bacteriocins and is used in a wide variety of food products as an effective factor in the inhibition and even elimination of foodborne pathogens and spoilage microorganisms in dairy, meat, fish, seafood, fruits, vegetables, cereals, and their derivatives [18,19,20,21,22,23,24].

When applied as biopreservatives in the food industry, bacteriocins (alone and in combination with other natural preservatives, such as bacteriophages) are shown to effectively reduce the presence of spoilage microorganisms and foodborne pathogens [25]. For example, the industrial application of bacteriocins and bacteriocinogenic strains is a clear proof that they can contribute to the reduction of *Clostridium* spp. in dairy products and pasteurized eggs [20,26]; *Listeria monocytogenes* in fish, dairy, and meat products [27]; and vancomycin-resistant enterococci in different food products [28]. A clear advantage of bacteriocins compared with other antimicrobials and conventional sanitization, conservation, and sterilization processes is their selectivity and antimicrobial spectrum of activity [2]. Bacteriocins normally possess a rather narrow spectrum of activity and are carefully selected for a specific purpose. They can inhibit selected spoilage and foodborne pathogens, while microorganisms associated with technological purposes, such as starter cultures, remain unaffected [29]. In contrast, most of the sanitizing, conservation, and sterilization processes kill practically all vegetative cells and some spore forms of any microorganisms present in the food product, including technologically important starter, adjunct, or probiotic cultures.

## 3. Effect of Environmental Factors on the Bacteriocin Effectiveness

Interactions between bacteriocins and other components of the food matrix are a topic that has been investigated by some researchers but still needs much more attention. A research approach focused on bacteriocin–food matrix interactions will give the ability to predict the efficacy of studied bacteriocins in a specific food matrix. It is known that most bacteriocins are cationic molecules and are reported as being amphipathic [5,7]. Based on their chemical specificity, bacteriocins can interact with target organisms. Similarly, they can also have specific behaviors in the food environment. Components of the food system can inhibit or promote bacteriocin activity, and these potential interactions need to be evaluated for any new bacteriocins and their application in specific food systems. A significant number of papers report on the high efficacy of nisin in dairy products [30,31,32], and the industrial application of nisin in the biopreservation of different soft cheeses, dairy spreads, and other dairy products has been explored [33,34,35]. However, nisin is less effective in meat products [34,36,37,38]. It has been suggested that the interaction of nisin with lipids from meat products can “trap” the bacteriocin and reduce its efficacy. However, other bacteriocins are effective in a meat environment. For instance, *Lactiplantibacillus plantarum* (formerly *Lactobacillus plantarum*) 423, producer of plantaricin 423, a class IIa bacteriocin, was studied as a promising biopreservative culture in the preparation of different types of salami [39]. Results showed that *L. monocytogenes*, used as a test organism (sensitive to plantaricin 423), was significantly reduced to the level of no detection when *Lb. plantarum* 423 was used as a starter–adjuvant culture in the preparation of salami. However, the application of a non-bacteriocinogenic mutant of *Lb. plantarum* 423 did not affect the growth of *L. monocytogenes* [39]. Numerous authors have evaluated the application of bacteriocins or bacteriocin-producing strains in meat preservation, and these works suggest a beneficial effect of bacteriocins as antimicrobials on the reduction of foodborne pathogens in the final products [37,38,40,41,42,43,44,45,46,47,48,49].

## 4. Bacteriocins: Safety Is the Priority

Another reasonable question is related to the appropriate choice of the cell line used for evaluating the cytotoxicity of bacteriocins. Most studies use Caco-2 as a model cell line for determining the cytotoxicity of different molecules, including bacteriocins. However, are cancer cell lines really a good model? Vero cells were utilized in several studies on the cytotoxicity of different bacteriocins [12,13,33,50,51,52,53], and the human hepatocellular carcinoma cell line, Huh7.5, has also been used [53]. The pharmaceutical industry recommends specific cell lines to determine the cytotoxicity of different pharmaceutical molecules, and the determination of bacteriocin cytotoxicity should adhere to the same or at least equivalent standards.

The term ‘cytotoxicity’ generally refers to a broad and ill-defined meaning in the drug discovery and pharmaceutical development industry. In the case of *in vitro* cell culture assays, for a compound or treatment to be considered cytotoxic, there needs to be evidence that it can interfere with cellular attachment, significantly alter morphology, adversely affect cell growth rate, or cause cell death [54,55]. Various assays have been developed and used for measuring viability or cytotoxicity *in vitro*, including classical dye inclusion or exclusion and colony formation assays [55,56,57,58]. Despite the variety of assays available for determining cytotoxicity not only for bacteriocins but different pharmaceutical and cosmetic products, well-accepted standards are fact and have been established [55,59].

While bacteriocins are generally considered to be nontoxic for mammalian cells, some examples easily poke holes in this assumption. Cytolysin is an antimicrobial peptide produced by several strains of *Enterococcus* spp. and showed toxicity at high concentrations [60]. Cytotoxicity needs to be considered as a milestone test when the safety of bacteriocins is being evaluated prior to their use in the food industry or in human or veterinary medicine. However, specific cytotoxicity against targeted cells can also be an advantage and should be explored for designing drugs targeting specific mammalian cells, such as cancer cells [61,62,63].

There was no reported evidence of acute toxicity when nisin was orally administered at 1 g/kg in rats [64]. However, Vaucher et al. [65] reported on possible toxicity in mice as evidenced by histological changes in the spleen, skin, and liver after exposure to 0.825 mg/kg nisin for 21 days. Moreover, the authors described that these histological changes were associated with specific, possibly inflammatory processes that it will be difficult to differentiate between the effect of purified nisin and the high salt content of commercial nisin (Nisaplin^®^) used in the mentioned study [65]. Extrapolating this to humans and considering that the weight of an average man is approximately 70 kg, this would be equivalent to a daily uptake of 50–70 g of nisin, an amount that is much higher than the realistic potential intake of nisin from different food products where nisin was applied as a biopreservative. Sahoo et al. [66] reported that the LD50 of the bacteriocin TSU4, which is produced by *Ligilactobacillus* (formerly *Lactobacillus*) *animalis* TSU4, is higher than 200 mg/kg when orally administered to mice. Moreover, after oral administration for 21 days at 0.5 mg/kg, the bacteriocin TSU4 showed neither mortality in mice nor significant changes in physiological conditions and immunological markers, an argument for the absence of a toxic effect for the studied bacteriocin [66].

Additional arguments for the safety of pediocin N6, another bacteriocin, were reported by Marlida et al. [67], who administered up to 20,000 mg/kg of pediocin N6 in mice. Lactocin 160 is a bacteriocin produced by *Lacticaseibacillus* (formerly *Lactobacillus*) *rhamnosus* 160 with activity against different pathogens associated with bacterial vaginosis. Lactocin 160 was intravaginally applied in rabbits at 18 mg per treatment, and no evidence of acute toxicity was observed. Furthermore, in vitro and in vivo safety evaluation experiments showed that lactocin 160 is not associated with any severe irritation of vaginal epithelial tissue [68]. A bacteriocin produced by *Enterococcus faecalis*, enterocin AS-48, showed no toxicity when intraperitoneally administered to mice (100 g/mouse, corresponding to 5 mg/kg) every 8 h over a 2-day period, and no skin sensitization or allergic contact dermatitis was observed [69].

However, studies that would seem to show the opposite of the above-mentioned results have also been reported: nisin and pediocin PA-1 were cytotoxic when applied at higher concentrations against Vero cell lines [70,71]. Moreover, it was reported that a semi-purified bacteriocin produced by *Lb. plantarum* ST8SH was highly cytotoxic at a concentration of 25 μg/mL, but not at 5 μg/mL [72].

Since bacteriocins are polypeptides (small-sized proteins), it is assumed that proteolytic enzymes in the stomach and other parts of the intestinal tract can hydrolyze them, rendering them inert. However, what about the upper regions of the GIT? What about the protective effect from various components of food matrixes? All these are subjects that merit further attention and deeper analyses.

The sensitivity of bacteriocins to the effects of proteolytic enzymes present in the GIT of humans and other animals can be discussed in terms of both positive and negative characteristics. On one side, when applied in the biopreservation of food products, leftover residues of bacteriocins can be digested by proteolytic enzymes in the GIT, lowering the risk of cytotoxicity. However, if the target of bacteriocins is pathogens within the GIT, several barriers may be involved and affect their stability and biological activity. It is well-established in different in vitro and in vivo studies that orally administered bacteriocins and even those produced in situ are inactivated or digested by proteolytic enzymes naturally present in the GIT [73,74,75]. Kheadr et al. [76] reported that pediocin PA-1 was stable in the stomach. However, it was degraded when exposed to the conditions of the small intestine. It has been suggested that class I bacteriocins may be more resistant to proteases compared with class II bacteriocins due to undergoing extensive post-translational modifications [77,78]. It was reported that nisin can remain partly stable when exposed to gastrointestinal fluids; however, bioengineered bacteriocins or encapsulation can improve stability [79]. Nisin bioengineering of the C-terminal region can improve the bacteriocin’s activity and stability in the presence of proteolytic enzymes [80,81]. Since most bacteriocins are expected to perform at the end of the small intestine and in the colon, encapsulation was suggested as a potential approach for the effective protection and controlled delivery of these molecules [17,82,83,84].

The immune response to the administration of bacteriocins is considered an essential part of evaluating the safety of antimicrobial peptides and other food additives or pharmaceutical preparations [85,86]. Pediocin AcH (also known as pediocin PA-1) [87] and TSU4 [66] were shown to be non-immunogenic. However, when administrated over more extended periods of time, a nisin-containing preparation (that also contains milk proteins and salt) can be associated with a significant increase in macrophages and monocytes isolated from the peripheral blood [88]. After the administration of pyocin S5, low levels of antibodies were recorded in the serum of the test animals [89,90].

As part of the safety evaluation of bacteriocins, it is important to observe no adverse effects on the integrity of tight junctions or cell-to-cell adhesion [91]. Different cell lines mimicking the GIT or the intestinal mucosa have been utilized in the evaluation of the effect of bacteriocins on tight junctions, including HCT-8, HT29, and Caco-2 [92,93,94,95]. According to Belguesmia et al. [91], nisin and enterocin S37 can be considered safe since no significant reduction in epithelial integrity was observed when both bacteriocins were supplemented at 10 μg/mL to Caco-2/TC7 cells. Moreover, when Caco-2 cells were treated with 2 μg/mL of divercin AS7 for 24 h, no changes in the integrity of the cells were observed [96]. In a similar experiment, the interaction between Caco-2 cells and plantaricin A showed that the bacteriocin can protect the cells’ integrity and tight junctions [94].

## 5. Interactions between Bacteriocins and Environmental Factors

The role of environmental factors (temperature, pH, presence of chemicals) has been the subject of several studies related to bacteriocins produced by various LAB [44,97,98,99,100,101]. In addition to evaluating optimal conditions for bacteriocin’s action, these reasonably simple methods can predict the efficacy of new bacteriocins and substantially reduce research costs and time. On the other side, the specificity of a bacteriocin’s adsorption to and effect on target pathogens is very useful information to know which foodborne pathogens and spoilage organisms can be targeted during the biopreservation processes.

Bacteriocin adsorption to target cells can be based on the recognition of specific receptors and charge-based interactions or interference with hydrophobic surface molecules and is considered the first step of the antibacterial mode of action (for a review, see, for instance, Drider et al. [102], and for a study of model interactions, see Soliman et al. [103]). To penetrate the cell membrane and disturb cellular integrity, some, if not all, bacteriocins need to be effective in their recognition of specific receptors or/and to have strong physicochemical interactions with target cells. This process may include a specific target such as lipid II, recognized by lantibiotics, and some antimicrobial proteins of eukaryotic origin (for review, see Grein et al. [104]). However, a lack of interaction with lipid II is reported for some lantibiotics as well; moreover, different groups of lantibiotics have distinct interactions with lipid II as a target (comprehensively reviewed by Wang et al. [105]). Other targets (recognition sites, “anchoring molecules”) were reported for different bacteriocins, such as mannose phosphotransferase for pediocin PA-1, a class IIa bacteriocin [106]. These complex processes are influenced by several factors, including pH, temperature, and the presence of chemical agents. All of these factors can influence physiological conditions and the integrity of the cell membrane and, consequently, can influence the interaction of bacteriocins with target receptors or their direct contact with the cell membrane.

The in-depth evaluation of the influence of environmental factors on the adsorption of bacteriocins to target cells can be a valuable tool in predicting the efficacy of bacteriocins, not only in food biopreservation processes but also in the primary selection of potential therapeutically active antimicrobial agents for human and veterinary medicine application.

## 6. Application of Bacteriocins

How can we apply bacteriocin(s) in food biopreservation processes? There is consumer demand for better food products: products with a longer shelf life, without chemical preservatives, more natural ingredients, etc. Food retail businesses provide choices to the consumers and offer food products that fit into the concept of “bio products” or “organic products”. However, the final choice of consumers is often driven by the price of the product. In addition, the safety of “bioproducts” or “organic products” is debatable and has already been the subject of discussion and evaluation [107,108]. If applying natural biopreservatives, including and focusing on bacteriocins, increases the final product’s price, will this food product be competitive on the retail market? This is why most research groups agree that applying highly purified bacteriocins is not an economically feasible option. A more realistic approach is to apply partially purified bacteriocins or guarantee in situ bacteriocin production by applying bacteriocin producers as starter or adjunct cultures in the fermentation process (Table 1).

The perfect scenario then is to develop one culture that will be responsible for solving all the needs from the food technology point of view (such as to be an excellent starter culture) that, at the same time, can be a producer of antimicrobial metabolites and will guarantee food safety and microbial quality by inhibiting potential spoilage and foodborne pathogens. This will be a perfect scenario, but it is unrealistic and unlikely to be achievable, almost the same as the desire of alchemists to find a *Panacea*, a universal drug for all illnesses. Starter cultures and biopreservative cultures have different roles in fermentation processes. Most of the LAB starter cultures play a role in the acidification of their environments [128,129] and produce a variety of enzymes (including proteolytic and lipolytic enzymes), particularly important in the preparation of fermented meat products [130,131]. In dairy products, proteolytic enzymes produced by LAB starter cultures can be actively involved in the reduction of allergenicity by hydrolysis of specific proteins, particularly caseins [132,133]. There is ongoing research on the use of LAB starter cultures’ enzymatic systems to reduce gluten in fermented cereal products [134]. Bacteriocins can be important players in food safety. However, they need to be produced at reasonable levels, especially considering the temperature during the preparation of food products, availability of nutrients for the bioprotective LAB culture, etc. They must exhibit their activity without being affected by proteolytic enzymes produced by the starter cultures [34].

There are several other issues that are worth mentioning. It is important in cases where the bacteriocinogenic culture will be applied as an adjunct starter culture that the specific culture does not influence the organoleptic characteristics of the final product while also being safe for application in food and feed products. Safety measures are essential and need to include physiological and biochemical tests for antibiotic susceptibility, hemolytic activity, and potential production of biogenic amines [135,136]. The presence of virulence-related genetic determinants should be addressed as well. Concerns about the possible transfer of those virulence genes have been the subject of several studies and models. It should be mentioned that Suvorov [137] reported on the low probability of possible horizontal gene transfer between enterococci and other LAB or pathogens in the GIT. However, safety issues related to the presence of virulence factors in the starter and biopreservative cultures need to be evaluated as well, bearing in mind that specific microbial cultures will be exposed to very different conditions, and it is the researcher’s responsibility to guarantee that none of them are carrying virulence determinants in any known forms.

Cereal-based fermented products are popular and consumed all around the world. The food industry has been adapting existing traditional products or developing new cereal-based food products (fermented or not) while taking into consideration consumer preferences. The Balkan Peninsula is known for one of its traditional fermented cereal-based beverages, boza [138]. This product is traced back to the ancient times and is highly appreciated by different ethnic groups because of its nutritional value and as a part of traditional medicine in the region [138,139]. Boza has been described as a source of numerous bacteriocin producers [138,139,140,141,142,143]. Specific antimicrobial characteristics for some of the boza-derived LAB and their bacteriocins have even been linked to antiviral, anti-*Mycobacterium tuberculosis*, and anti-fungal activity [45,144]. Interactions between bacteriocins produced by LAB, part of the boza starter culture consortium, and spoilage microorganisms or foodborne pathogens were evaluated, and the role of antimicrobial proteins was highlighted [46,145].

To evaluate the potential of two different bacteriocinogenic strains (*E. faecium* ST88 and *Enterococcus mundtii* CRL35) as potential biopreservative cultures, Pingitore et al. [146] performed a model study of cheese preparation and applied both strains as adjunct cultures. In a series of preliminary tests, the authors have shown the behavior of the studied bacteriocinogenic strains in laboratory conditions relating to their interactions with *L. monocytogenes* (the target foodborne pathogen in this project). In addition, the behavior of bacteriocins and interactions with the target organism (*L. monocytogenes*) were evaluated at different temperatures and pH, and in the presence of dairy ingredients and commonly used additives to better understand and predict effectiveness in upcoming in situ experiments. Pingitore et al. [146] clearly showed that the level of bacteriocin production (6400 AU/mL for *E. faecium* ST88 and 25600 AU/mL for *E. mundtii* CRL35), recorded against *L. monocytogenes* 426, can play a critical role in controlling the targeted foodborne pathogen. We need to acknowledge that the level of bacteriocin production in a commercial MRS medium and a dairy environment (in milk) cannot be the same, and this caveat has been discussed in several papers [146,147,148].

Effective antimicrobial action against different strains of the target pathogen is necessary to ensure that the proposed bacteriocin can protect the food product. The selected bacteriocinogenic strain and bacteriocin should be highly effective against targeted pathogens but, at the same time, should not inhibit the growth of the beneficial microbiota. This has been discussed and pointed out by several research papers. Pingitore et al. [146] performed experiments on the adsorption of bacteriocins (produced by *E. faecium* ST88 and *E. mundtii* CRL35) to *L. monocytogenes* under different conditions, including various temperatures, pH, and in the presence of cheese-making additives, and the *E. mundtii* CRL35 bacteriocin was shown to be superior as compared with the activity of the bacteriocin from *E. faecium* ST88. We need to keep in mind that for any bacteriocin to act as an antimicrobial, the first step will be to adhere to the surface of the target strain. This may depend on the recognition of a specific receptor or can be a nonspecific adherence to the cell wall [7]. For that specific bacteriocin produced by *E. mundtii* CRL35 [146], the technological conditions of the cheese-making procedure, including conditions such as low pH and production, maturation, and refrigeration temperatures, were optimal for bacteriocin adsorption to *L. monocytogenes*. This study presents possible positive results for the control of *L. monocytogenes* by bacteriocins produced by *E. faecium* ST88 and *E. mundtii* CRL35 with potentially better performance by the bacteriocin mundticin (produced by *E. mundtii* CRL35).

## 7. How Effective Can the Application of Partially Purified Bacteriocins Be?

Nisin is a great example of the successful application of bacteriocins (lantibiotic, class I, classification by Heng et al. [149]). It has been nearly one century since the discovery of nisin, and the long history of its safe food-associated applications can be attributed to its extremely low cytotoxicity, similar to that of NaCl [150], and its sensitivity to digestive proteases and absence of any influence on the sensory properties of food [151].

Commercial applications of nisin are regulated by the European Union Food Safety Authorities, where it is licensed as a food preservative (E234). The FAO/WHO Codex Committee on milk and milk products has authorized nisin to be applied as a food additive in processed cheeses, with a limit of 12.5 mg (as calculated for pure nisin) per kilogram of the product [152]. However, commercial nisin is a partially purified industrial preparation. The commercial preparation of nisin follows the strict rules and regulations developed by international and national authorities following the recommendations of the FAO and WHO. When we apply semi-purified commercial bacteriocin preparations, attention must be given to the composition of the supporting components and ballast ingredients.

Moreover, Vaucher et al. [65] pointed out the fact that some histological changes after application of Nisaplin^®^ can lead to specific inflammatory processes, most probably not due to the effect of nisin itself but from the high salt content in the industrial formulation (Nisaplin^®^) [65]. In commercial preservation processes, different salts and milk residues in the Nisaplin^®^ formulation can be responsible for the observed side effects. In addition, the application of nisin is a practice in the biopreservation of pasteurized eggs, canned food, some meat, and bakery products [30,32]. However, when applied to food products, bacteriocin(s) and bacteriocinogenic formulation ingredients (such as bacteriocinogenic starter cultures, protective cultures, etc.) need to be clearly stated on the label to alert the consumers to the potential presence of allergenic ingredients (dairy or salts) in the product.

## 8. Limitations of Use of Bacteriocin Producers

The application of bacteriocin producers as adjuncts or starter cultures can be considered a perfect scenario for biopreservation processes, which has been proposed by several research papers suggesting that specific LAB can provide technological (for performing biotransformation of raw products to a fermented food product) properties, in addition to biopreservation properties, producing different bioactive molecules and contributing to the food safety of the final product [34,72,131,133]. However, relatively few have been implemented in practical industrial processes. The principal problem may be an absence of communication or collaboration between academia and industry. Scientific problems related to applying LAB as live cultures with biopreservative properties can also be related to the fact that some bacteriocin producers cannot meet the criteria for safety assessments. The appropriate regulatory governmental organization controls the applications of starter and adjunct cultures to guarantee the safety of food products regionally and internationally. The WHO, EFSA, and FDA are some examples of these agencies.

Bacteriocin production is reported in different LAB [7]. However, not all LAB are considered safe. A good example is found in species belonging to the genera *Streptococcus*, where the GRAS status is granted only for *Streptococcus thermophilus*. The rest of the *Streptococcus* spp. are not considered safe, and some are serious human and animal pathogens and are even related to some types of cancers, as reported for some *Streptococcus bovis* strains [153,154,155]. *Enterococcaceae* is yet another good example. Some species are well-known and have been used for centuries as starter cultures, especially in the Mediterranean region, in the production of different types of cheeses and salami [156]. On the other hand, numerous *Enterococcus* spp. can be directly linked to nosocomial infections, and vancomycin-resistant *Enterococcus* is a serious health problem [157]. Some infections were directly linked to medical cases related to endocarditis, bacteremia, and intra-abdominal, pelvic, urinary tract, and central nervous system infections [158]. The presence of vancomycin-resistant enterococci, most probably related to the use of avoparcin as a feed additive in animal husbandry, is a significant threat to public health since glycopeptides are considered to be the last line of drugs in the treatment of enterococcal infections [159,160,161,162,163]. Antibiotic resistance alone cannot explain the virulence of enterococci. The process is more complex, and most infections follow a common sequence of events involving colonization and adhesion to host tissues, invasion of the tissue, and resistance to both nonspecific and specific defense mechanisms of the host. The pathogen must produce pathological changes either directly by toxin production or indirectly by inflammation [164].

Studies of the presence of virulence factors and antibiotic resistance genes have been conducted for different LAB [136,165,166] and showed that some “GRAS species” (although the GRAS status is usually assigned to a strain) can carry some of these genes, compromising their safety. The general question, then, is whether the bacteriocin producer can be defined as safe. Comprehensive safety evaluations need to be performed for any strain before application in a specific part of the food production process to guarantee the safety of the consumers.

Most LAB are commonly considered safe based on their long history of use in different fermented food processes [167]. However, some LAB are reported as pathogens (some reports are Land et al. [168], Kulkarni and Khoury [169], Salminen et al. [170], Karime et al. [171], and an old but comprehensive review: Aguirre and Collins [172]) and food spoilage microorganisms (for instance, Kalschne et al. [173] and Andreevskaya et al. [174]).

A different problem is related to a more practical issue. When working with bacteriocin production, most of the studies have been performed using a very rich growth medium such as MRS [97,99,100,148]. Several studies were reported on the optimization of bacteriocin production, applying simple approaches such as exclusion or replacement of some media components, or building sophisticated mathematical models and following the effect of different constituents of the growth medium and cultivation parameters on bacteriocin production [97,99,100,148]. Different byproducts have been proposed as alternatives for the low-cost production of bacteriocins of varying types and origins [97,148]. The question, however, remains: will LAB be able to produce sufficient levels of bacteriocins and at the specific desirable amount to effectively protect against food spoilage? Will bacteriocin be able to reach the entire food system to guarantee its efficacy? Studies have shown that some of these problems can be considered solved, as the authors reported on the efficacy of bacteriocins and their production in situ [175]. However, we cannot always expect high bacteriocin expression levels across different food environments. The concentration and availability of nutritional components can be limited in addition to competition with other microorganisms. The problem, however, is that many studies are not adequately planned and executed, as there is an obvious conflict between optimal conditions for bacterial growth, the production of bacteriocins, and the conditions needed when performing model studies. For instance, many studies were conducted under refrigeration conditions. Under these conditions, *L. monocytogenes* can multiply, but this is far below the temperature limits for LAB, and we cannot expect LAB to produce bacteriocins if they experience low-temperature stress.

In summary, when planning to apply specific bacteriocinogenic LAB as live adjunct cultures, we need to be sure that the fermented food product will provide specific nutrients related to bacterial growth and antimicrobial protein production. Additionally, the technological temperature of the production of food products must be in accordance with the specific temperature needs of the bacteriocin-producing LAB, neither very low (as refrigeration) nor very high (as part of cooking or sterilization of the product).

## 9. Spectrum of Activity: Kill Only Bad and Not Good

One of the biggest advantages of bacteriocins as antimicrobial agents is their relatively high selectivity [5]. Antibiotics are defined as chemotherapeutic agents with activity against microorganisms (bacteria and fungi) and protozoa [176]. Antibiotics are highly effective in controlling pathogenic bacteria. However, they often kill the beneficial microbiota as well. Cases of antibiotic-related diarrhea as a consequence of the treatment of clinical cases in patients are a serious problem in hospitals. Clinical practices recommend the application of vitamins from group B, supporting beneficial microbiota treatment, enforcement with selected probiotics, and, in some cases, the consumption of traditional fermented food products rich in LAB [177].

The application of antibiotics is strictly controlled in animal feed as growth promoters and is authorized only as therapeutic agents for the treatment of sick animals and not as prophylactic treatment in veterinary practice. The uncontrolled application of antibiotics in veterinary practice in the past is one of the well-recognized reasons for the rise of antibiotic resistance. According to the European Commission in 2005, more than 10 million tons of antibiotics have been released into the biosphere since the start of commercial production. This has exerted a very strong selection on the development of resistant strains. Resistance may be inherent to a bacterial genus or species but may also be acquired through the exchange of genetic material, mutations, and the incorporation of new genes [178]. Teuber et al. [179] suggested that starter cultures and probiotics may serve as vectors in the transfer of antibiotic resistance genes. Such a transfer has been documented in other bacterial groups by Levy and Marshall [180].

The specific influence of bacteriocins on the GIT microbiota needs to be considered as an important issue, since long-term application may have a negative effect on diversity and consequently on function and may even be associated with different diseases, including inflammatory immune diseases, functional bowel disorders, insulin resistance, and obesity [181]. The effects of bacteriocins on changes in the diversity of intestinal microbiota in different regions of the GIT were reported by Kheadr et al. [76], Le Blay et al. [182], Guinane et al. [183], and Le Lay et al. [184] by different in vitro models. Treatment with nisin and pediocin PA-1 did not result in significant changes in the commensal GIT microbiota for in vitro evaluations [182,183,184] and in vivo model studies [185,186]. On the other hand, the opposite was reported in an in vitro study [187], where lacticin 3147 (class I bacteriocin) induced a shift in the microbiota from Firmicutes to Proteobacteria.

Umu et al. [175] have discussed the production of bacteriocins as part of the arsenal of the probiotic potential of LAB in preventing the growth of pathogens in gut environments. The authors evaluated the potential of five bacteriocin-producing strains of LAB (producers of sakacin A; pediocin PA-1; enterocins P, Q, and L50; plantaricins EF and JK; and garvicin ML) and their isogenic nonproducing mutants for probiotic potential in an animal model via 16S rRNA gene analysis. When combined with appropriate pre-selection analysis based on an evaluation of pH, temperature, and environmental conditions, this can be the ideal approach to making the right choice when selecting potentially effective bacteriocinogenic strains.

In an animal model study on rats, Bernbom et al. [185] reported on the use of *Lc. lactis* (nisin producer) or purified nisin that increased the presence of bifidobacteria and decreased the number of enterococci in fecal samples. However, in a different study on bacterial cultures associated with human microbiota, nisin A and nisin Z showed inhibitory effects on bifidobacteria and lactobacilli. When the effect of pediocin was evaluated, no changes in the two mentioned microorganisms’ presence were observed [188]. Similar results were reported by Dabour et al. [186] when they evaluated the effect of a bacteriocin on bifidobacteria and lactobacilli in a model animal study in mice. Moreover, Dobson et al. [189] reported that oral administration of *Lc. lactis* DPC6520, a lacticin 3147 producer, did not affect diversity and proportions within the microbiota of the distal colon. Similar arguments for the effect of bacteriocin Abp118, produced by *Ligilactobacillus* (formerly *Lactobacillus*) *salivarius* UCC118As, were presented by Riboulet-Bisson et al. [190], showing no significant influence on the proportion of the general microbial communities of mouse gut microbiota, with a significant decrease only in Spirochetes and Firmicutes. In a similar experiment using a mouse model, Murphy et al. [191] reported that when applied, some specific bacteriocinogenic strains did not significantly impact Firmicutes but may increase the presence of Bacteroidetes and Proteobacteria and reduce representatives of Actinobacteria.

In a murine animal model, administration of bacteriocinogenic *Lb. salivarius* UCC118 showed an increase in Peptococcaceae and a reduction in the proportion of Riknelleaceae and Porphyromonadaceae [192]. Previous examples are evidence that the bacteriocinogenic strains and bacteriocins themselves can impact the functional diversity of the colonic microbiota. However, the impacts of bacteriocins and bacteriocin-producing strains are not yet fully understood, and additional in vitro and in vivo studies need to be designed, including the application of different strains and bacteriocins together with different animal models.

Even if several studies demonstrated the beneficial role of bacteriocins in the control of spoilage microorganisms and foodborne pathogens, pointing to an important role of the narrow spectrum of activity of bacteriocins and their effectiveness against targeted species, some authors have raised their concerns about the application of bacteriocins associated with the fact that these antimicrobials may be too narrow in their spectrum of activity [23]. Moreover, bacteriocins can be trapped by lipids due to their hydrophobic nature, which are naturally present in many food products [193]. From a general point of view, predicting what specific spoilage and/or foodborne pathogen should be targeted and which bacteriocin will have to be applied will be difficult.

Mills et al. [193] mentioned that the distribution of bacteriocins into the food matrix can be yet another limiting factor diminishing their effectiveness. However, targeting specific areas where spoilage of foodborne pathogens can be present in high numbers should be considered as an additional strategy for the application of bacteriocins as effective biopreservatives. Zhou et al. [194] suggested that plantaricin BM1 can be spread on the surface of hams and, in this way, can be more effective in inhibiting *L. monocytogenes*. Incorporating bacteriocins into the packaging material, from which they can be released and effectively inhibit targeted spoilage or pathogens, is yet another approach presently under investigation [195].

Although some may see it as a potential disadvantage, most authors agree that bacteriocins have the advantage of being rather selective in their spectrum of activity and being able to inhibit particular species. Selectivity is an advantage as most bacteriocins can be applied in an appropriate system for the control of specific targeted pathogens while leaving other parts of the microbial population unhindered. Selectivity is a key feature of antimicrobial peptides that cannot be associated with antibiotics or chemical antimicrobials. Udompijitkul et al. [196] suggested the application of nisin for the control of germinated spores of *Clostridium perfringens* in a rich nutrient environment. Bartoloni et al. [197] evaluated the effect of nisin against clinical isolates of *Clostridium difficile* in comparison with vancomycin and metronidazole by minimum inhibitory concentration (MIC), minimum bactericidal concentration (MBC), and time–kill studies. According to Bartoloni et al. [197], nisin was more active than the other agents, with an MIC_90_ of 0.256 mg/L and strong bactericidal activity. They suggest that nisin may be a promising agent for the management of *C. difficile-*associated diarrhea. Application of bacteriocins for the control of *Clostridium* spp. without influencing the beneficial microbiota is just one example of a beneficial application of antimicrobial proteins from LAB.

## 10. Conclusions

Throughout the human history, food has always played an essential role. Traditions, religion, society, and family have always been and will continue to relate to or revolve around food. For centuries, preparing and preserving food commodities has been an empiric practice, transmitted from one generation to the next. The life sciences, established in the 18th and 19th centuries, have generated a collection of fundamental and applied knowledge over time. From a 21st-century perspective, however, the preparation of food products is a complex process, based on the involvement of biochemistry, microbiology, physics, chemistry, engineering, etc. Over the last century, the food preservation processes became better understood, and the role of different antimicrobial metabolites started to be clarified. Nowadays, consumers are not only satisfied by the nutritional values of food products but also pay significant attention to the safety and health-promoting properties of food commodities (Figure 1). In essence, food needs to be affordable, nutritional, safe, and health-promoting. The role of bacteriocins from LAB, as part of the larger family of antimicrobial peptides, has been proven by their contribution to the biopreservation processes. Bacteriocins can be clearly associated with controlling spoilage and foodborne pathogens, extending shelf life, and replacing chemical additives and preservatives. The more we extend our knowledge of bacteriocinogenic LAB and their role in fermentation and biopreservation processes, the more we discover that additional scientific questions will need to be addressed and answers provided. Currently, a solidly built scientific structure stands behind the application of bacteriocins in food and other areas, but this structure needs to be maintained and built upon in order to provide consumers with safe, nutritional, and beneficial food in the future.

Driven by scientific curiosity and demand from the food and pharmaceutical industries and both consumers and patients, research on antimicrobial proteins, including bacteriocins, is facing new challenges regarding an affordable cost of production and the partial purification of antimicrobial proteins to concentrations that are appropriate for specific applications. Additional post-production modifications can be an option for increasing the stability of bacteriocins, improving delivery to target sites in the case of both infections and the biopreservation of foods. Bioengineering of existing bacteriocins and replacing specific amino acids or regions is one possible way of modulating or increasing their spectrum of activity and extending their application areas. The abovementioned scientific challenges in the study of bacteriocins are representative of some of the challenges that still need to be addressed through academic and applied research of antimicrobial peptides.

## Figures and Tables

**Table 1:** Examples of some proposed food and feed-related applications for some of recently reported bacteriocins.

Bacteriocin	Producer	Area of Proposed Application	Reference
Bovicin HC5 and nisin	*Streptococcus bovis* HC5	Control of *Alicyclobacillus acidoterrestris* in fruit juices	[109]
Bovicin HC5	*Streptococcus bovis* HC5	*Clostridium tyrobutyricum*, a pathogen associated with spoiled mango pulp	[110]
Enterococin A	*Enterococcus faecium MMRA*	Control of *Listeria monocytogenes* in sliced dry-cured ham	[111]
Bacteriocin RSQ04	*Lactococcus lactis* CGMCC20699	Evaluation of activity against *Listeria monocytogenes* in model food system	[112]
Pyocin QDD1	*Pseudomonas aeruginosa* QDD1	Biocontrol of foodborne pathogens *Staphylococcus aureus* and *Bacillus cereus*	[113]
Bacteriocin LSB1	*Lactiplantibacillus plantarum* LSB1	Activity against *Staphylococcus argenteus* planktonic cells and biofilm	[114]
Bacteriocins ST20Kc and ST41Kc	*Enterococcus faecium* ST20Kc and ST41Kc	Control of *Listeria monocytogenes* and vancomycin-resistant entorococci	[115]
Bacteriocin LSX01	*Lacticaseibacillus paracasei* LSX01	Reduction of planktonic cells of *Staphylococcus aureus*	[116]
Bacteriocin CTC494	*Latilactobacillus sakei* CTC494	Anti-listerial activity in vacuum packaged cooked ham	[117]
Six bacteriocins	*Lacticaseibacillus casei* and *Lactiplantibacillus plantarum*	Activity against 8 different *Listeria monocytogenes* strains	[118]
Bacteriocins ST651ea, ST7119ea, and ST7319ea	*Enterococcus faecium* ST651ea, ST7119ea, and ST7319ea	Control of *Listeria monocytogenes* and vancomycin-resistant enterococci in GIT model system	[119]
Bacteriocin Sak-59	*Lactobacillus sakei* B-RKM 0559	Activity against meat spoilage bacteria strains of *Listeria monocytogenes, Staphylococcus aureus*, and pathogenic strains of *Serratia marcescens* and *Escherichia coli*	[120]
Lactocin 63	*Loigolactobacillus coryniformis* FZU63	Antimicrobial mode of action against *Shewanella putrefaciens*	[121]
Bacteriocin R23	*Lactiplantibacillus plantarum* R23	Anti-*Listeria monocytogenes* activity	[122]
Enterocin LD3 and Plantaricin LD4	*Enterococcus faecium* LD3 and *Lactiplantibacillus plantarum* LD4	Synergistic effect against *Staphylococcus aureus* subsp. *aureus* ATCC25923, *Salmonella enterica* subsp. *enterica* serovar Typhimurium ATCC13311, *Proteus mirabilis* ATCC43071, *Pseudomonas aeruginosa* ATCC27853, and *Escherichia coli* ATCC25922	[123]
Bacteriocins ST1607V, ST2104V and ST3105V	*Pediococcus acidilactici* ST1607V, ST2104V and ST3105V	Bactericidal mode of action against *Listeria monocytogenes* ATCC7644 and *Enterococcus faecium* ATCC19434	[48]
Bacteriocin BM1829	*Companilactobacillus crustorum* MN047	Reduction of *Escherichia coli* and *Staphylococcus aureus*	[124]
Enterocin K2B1	*Enterococcus faecalis* K2B1	Control of foodborne pathogens in dairy products	[125]
Bacteriocin OS1	*Enterococcus hirae* OS1	Anti-*Listeria* activity	[126]
Bacteriocins ST3522BG and ST3633BG	*Pediococcus acidilactici* ST3522BG and *Pedioccocus pentosaceus* ST3633BG	Anti-*Listeria* activity in silage fermentation models system	[127]

## Data Availability

Not applicable.
